# Transcriptional and Post-transcriptional Regulation of Organellar Gene Expression (OGE) and Its Roles in Plant Salt Tolerance

**DOI:** 10.3390/ijms20051056

**Published:** 2019-02-28

**Authors:** Pedro Robles, Víctor Quesada

**Affiliations:** Instituto de Bioingeniería, Universidad Miguel Hernández, Campus de Elche, 03202 Elche, Spain; probles@umh.es

**Keywords:** Salt stress, organellar gene expression (OGE), Arabidopsis, rice, mitochondrial transcription termination factors (mTERFs), pentatricopeptide repeat (PPR) proteins, DEAD-box RNA helicases (RHs)-containing proteins, RNA-recognition motifs (RRMs)-containing proteins, SIGMA FACTOR 5, PLASTID-SPECIFIC RIBOSOMAL PROTEIN 2

## Abstract

Given their endosymbiotic origin, chloroplasts and mitochondria genomes harbor only between 100 and 200 genes that encode the proteins involved in organellar gene expression (OGE), photosynthesis, and the electron transport chain. However, as the activity of these organelles also needs a few thousand proteins encoded by the nuclear genome, a close coordination of the gene expression between the nucleus and organelles must exist. In line with this, OGE regulation is crucial for plant growth and development, and is achieved mainly through post-transcriptional mechanisms performed by nuclear genes. In this way, the nucleus controls the activity of organelles and these, in turn, transmit information about their functional state to the nucleus by modulating nuclear expression according to the organelles’ physiological requirements. This adjusts organelle function to plant physiological, developmental, or growth demands. Therefore, OGE must appropriately respond to both the endogenous signals and exogenous environmental cues that can jeopardize plant survival. As sessile organisms, plants have to respond to adverse conditions to acclimate and adapt to them. Salinity is a major abiotic stress that negatively affects plant development and growth, disrupts chloroplast and mitochondria function, and leads to reduced yields. Information on the effects that the disturbance of the OGE function has on plant tolerance to salinity is still quite fragmented. Nonetheless, many plant mutants which display altered responses to salinity have been characterized in recent years, and interestingly, several are affected in nuclear genes encoding organelle-localized proteins that regulate the expression of organelle genes. These results strongly support a link between OGE and plant salt tolerance, likely through retrograde signaling. Our review analyzes recent findings on the OGE functions required by plants to respond and tolerate salinity, and highlights the fundamental role that chloroplast and mitochondrion homeostasis plays in plant adaptation to salt stress.

## 1. Introduction

Soil salinity severely affects plant development and growth and leads to yield losses. The high concentration of salts causes ionic imbalance and osmotic stress. Accordingly, salinity alters the ionic homeostasis of plant cells by causing toxic excess of sodium (Na^+^) and a deficiency of ions, such as potassium (K^+^) and Ca^+2^ because Na^+^ in excess disturbs the uptake of these cationic nutrients [1]. High Na^+^ content in the cytosol can also inhibit enzymatic activity, and can even interfere with protein surface charges by destabilizing molecular interactions. Apart from Na^+^, high levels of other ions, such as Cl^−^, Mg^2+^, SO_4_^2−^, or HCO^3−^ in the cytoplasm, can also contribute to salt toxicity [1]. To deal with the ionic and osmotic stresses imposed by salinity, plants have developed throughout their evolution several adaptive strategies [2].

A bigger world population and its associated food demand have meant having to increase the area dedicated to agricultural use and to develop ever-growing intensive and productive agriculture. Along these lines, the world’s human population is expected to reach more than 9 billion by 2050, and according to FAO 2011, global food production will need to increase by about 70% to match this population growth [1]. To face these demands, salinization of arable soils poses a serious threat. Accordingly, losses in the world’s agricultural production caused by the salinization of arable land are estimated to be around 12 trillion dollars per year, with more than 800 million hectares with high salinity levels [3]. This accounts for approximately 6% of the world’s land and affects one third of the world’s cultivated land area [4]. Therefore, it is necessary to advance in improving tolerance to crop salinity. One of the main strategies developed to avoid yields being lost to soil salinization is the selection of crop varieties that display enhanced tolerance to salinity. The development of these more halotolerant varieties requires unraveling the cellular, physiological, genetic, and molecular mechanisms that underlie plant salt tolerance. This is a difficult task, as plant salt tolerance is usually a genetically complex trait that is frequently modulated by different biosynthetic and signaling pathways. Crosstalks among these pathways have been reported under salinity conditions [5].

Abiotic stresses can be sensed by different plant cellular compartments, including mitochondria and chloroplasts [6], which are organelles with double membranes and with their own genomes. Cellular respiration in mitochondria (ATP production through oxidative phosphorylation) and photosynthesis in chloroplasts (the process by which plants produce organic substances from carbon dioxide and water using light energy from the Sun) are two vital processes to maintain life on our planet. The synthesis of essential biological molecules, such as nucleotides, amino acids, lipids, and vitamins, also takes place in these organelles [7,8,9,10]. Therefore, perturbation of chloroplast and mitochondrion homeostasis by endogenous or environmental cues may severely compromise plant growth and development. In line with this, chloroplasts are one of the organelles principally affected by salinity, which leads to lower carbon fixation rates and increased reactive oxygen species (ROS) levels [11]. An impaired plastid or mitochondrial function is communicated to the nucleus through retrograde signaling, and the activity of these organelles adjusts to cope with adverse environmental conditions. One way of achieving this is by modifying the activity of the nuclear genes that encode the chloroplast or mitochondrial proteins required for plants to adapt to stress [12,13]. Nonetheless, very little is known about the role that chloroplasts and mitochondria play in the response to abiotic stress in general, and to salinity in particular.

Chloroplast and mitochondrial genomes are the relics of those free-living prokaryotic organisms that invaded or were swallowed by a primitive eukaryotic cell with which they established an endosymbiotic relationship [14,15,16]. In the course of evolution, the number of genes in the genomes of these endosymbionts was drastically lowered, most of which were transferred to the nuclear genome. In this way, the gene functions needed for these organelles to properly function do not exclusively lie in their genomes because they depend largely on other functions located in the nucleus. This implies that a close coordination of the gene expression between nuclear and organellar genomes must exist. Along these lines, the vast majority of the proteins located in chloroplasts and mitochondria are translated in the cytosol and imported into these organelles as they are encoded by the nuclear genome. Accordingly, most studies about the effect of salinity on gene expression have focused on analyzing nuclear genes [5]. Proteomic and bioinformatic studies have indicated that chloroplast and plant mitochondrion proteomes, respectively, contain some 3000 and 2000 proteins [17,18]. In stark contrast, the genomes of these organelles harbor only between 100 and 200 genes that encode proteins involved in organellar gene expression (OGE, including transcription, RNA processing, and translation), photosynthesis in chloroplasts, and ATP production through oxidative phosphorylation in mitochondria. OGE regulation is crucial for plant growth and development. In chloroplasts, this is achieved mainly at the post-transcriptional level through the control of RNA translation, processing, splicing, decay, or editing [19]. Therefore, OGE must appropriately respond to changes that occur via development and environmental cues, including abiotic stresses, such as salinity. However, information about the effects that OGE disturbance has on plant tolerance to salinity is still limited. Notwithstanding, several works have recently reported thorough phenotypic and molecular characterizations of plant mutants, and to a lesser extent, of transgenic overexpression (OE) lines affected in nuclear genes involved in OGE in plastids or mitochondria, which exhibit altered sensitivity to salt stress. These genes regulate the expression of organellar genes at both the transcriptional and (mainly) post-transcriptional levels. Our review focuses on the results taken from analyzing these mutants and OE lines, and consequently, on OGE functions required for plant salt tolerance and response. Together, they highlight the important role of chloroplast and mitochondrion homeostasis in plant adaptation to salinity.

## 2. Effects of Perturbed OGE on Plant Tolerance to Salinity 

As many of the genes involved in OGE reside in the nuclear genome, their mRNAs must be translated into the cytoplasm, and the proteins they code must be imported to chloroplasts or mitochondria. In this way, the nucleus can control the activity of these organelles, while the latter transmit information about their functional state to the nucleus by modulating nuclear activity according to these organelles’ physiological requirements [20]. Accordingly, perturbed plastid gene expression is signaled to the nucleus, and affects the expression of photosynthesis-associated genes. In line with this, OGE is one of the several plant retrograde signaling routes proposed [21,22,23].

In the last few years, dozens of salt-responsive nuclear genes encoding chloroplast- or mitochondrial-targeted proteins from different plant species have been reported, some of which are involved in gene expression in these organelles (reviewed in [24,25]). The analysis of several plant mutants, mainly in *Arabidopsis thaliana* (hereafter Arabidopsis) and rice, has revealed a connection between chloroplast and mitochondrial functions and the response to salt stress. Some of these mutants are affected in nuclear genes involved in OGE and display altered responses to different abiotic stress conditions, including salinity (reviewed in [20]). Examples of such are genes that encode: (a) mitochondrial transcription termination factors (mTERFs), pentatricopeptide repeat (PPR), and other RNA-binding proteins that post-transcriptionally regulate OGE through RNA metabolism (e.g., DEAD-box RNA helicases (RHs) and RNA-recognition motifs (RRMs)-containing proteins); (b) the SIGMA FACTOR 5 (SIG5) required for plastid gene transcription; and (c) PLASTID-SPECIFIC RIBOSOMAL PROTEIN 2 (PSRP2).

### 2.1. Post-Transcriptional Regulation of OGE and Salt Tolerance

#### 2.1.1. Defective mTERF Mutants Show Altered Responses to Salinity

One of the gene families involved in OGE regulation is mTERF, for which several Arabidopsis mutants showing altered sensitivity to salt have been reported [26,27]. The mTERF proteins are targeted to chloroplasts or mitochondria, and contain a variable number of repeats of a motif dubbed “mTERF”, which is about 30 amino acids long. Plant genomes, mainly those of higher plants, contain larger numbers of *mTERF* genes than animals. Accordingly, 35 and 45 mTERFs have been reported in Arabidopsis and rice, respectively [28,29], and larger numbers have been proposed for other plants (e.g., 55, 56, and 62 for *Populous trichocarpa*, *Glycine max*, and *Malus domestica*, respectively [29]). This contrasts with the four mTERF (MTERF1 to 4) genes present in the nuclear genomes of metazoans. Both the expansion and diversification of plant mTERFs have been related to the tolerance and acclimation of plants to abiotic stress [27]. In vertebrates, mTERFs participate in mitochondria transcription termination, initiation, translation, and likely in mtDNA replication [30,31]. In plants, information about mTERF functions is rather limited. To date, very few mTERFs have been molecularly characterized; they all post-transcriptionally regulate the expression of chloroplast or mitochondria genes, and can bind DNA or RNA. Accordingly, Arabidopsis BELAYA SMERT/RUGOSA2 (BSM/RUG2) [28,32], mTERF15 [33], and *Zea mays* ZmTERF4 [34] are involved in organellar intron II splicing; mTERF6 has been proposed to participate in the maturation of the chloroplast isoleucine tRNA (*trnI.2*) gene [35] and the transcription termination of the plastid *rpoA* polycistron, which is important for transcription and translation in this organelle [36]. Along these lines, the first mTERF characterized in a photosynthetic organism, the *MOC1* gene of the unicellular green alga *Chlamydomonas reinhardtii*, is also involved in transcription termination in mitochondria [37,38]. Recently, Sun et al. [39] reported that mTERF4/COE1 (also known as BSM/RUG2) cooperates with the PPR protein GUN1 (GENOMES UNCOUPLED1) in plastid gene expression and retrograde signaling. Taken together, these results strongly support a role for plant mTERFs in regulating OGE.

As far as we know, a phenotype of altered sensitivity to salinity has been reported for *mda1* (*mTERF defective in Arabidopsis1*), *mterf6*, *mterf9*, *mterf10*, and *mterf11* mutants. Indeed, mutants *mda1,* affected in the *mTERF5* gene [40], and *mterf9* [41] are less sensitive to NaCl than the wild type, whereas *mterf6-2*, *mterf6-5* [42], *mterf10*, and *mterf11* [43] are salt-hypersensitive (Table 1). This suggests that mTERF5 and mTERF9 would negatively regulate Arabidopsis salt tolerance, whereas mTERF6, mTERF10, and mTERF11 would function as positive regulators of such tolerance [42]. Consistent with all this, the *mTERF10* and *mTER11* overexpression lines are more insensitive to NaCl than the wild type [43]. Interestingly, some of these mutants also exhibit an altered response to the abscisic acid (ABA) hormone, which plays a central role in the response and adaptation of plants to different abiotic stress conditions [44]. Accordingly, mutants *mda1* and *mterf9* are less sensitive to ABA than the wild type, whereas mutants *mterf6* and *mterf11* are ABA hypersensitive (Table 1) [40,41,42,43]. These results suggest that the enhanced or reduced tolerance of these mutants to salinity might be due, at least in part, to altered ABA signaling. The phenotype of an altered ABA response has also been reported for other mutants that exhibit enhanced or reduced sensitivity to abiotic stresses, besides salinity, which reinforces the fundamental role of this hormone in plant tolerance to adverse environmental conditions [20]. All the *mterf* mutants reported to show a salt stress phenotype are affected in mTERFs targeted to chloroplasts, and belong to either the “chloroplast cluster” (mTERF5, mTERF6, and mTERF9) or the “chloroplast associated-cluster” (mTERF10 and mTERF11) of proteins, which reveals the importance of chloroplast homeostasis for plant response to salinity [29].

In short, the analysis of the *mterf* mutants clearly indicates the involvement of mTERFs in the tolerance and acclimation of plants to adverse environmental conditions, especially to salt stress.

Apart from the above-reported results, Zhao et al. [59] made a systematic characterization of maize *mTERF* genes by bioinformatics and molecular biological approaches. By quantitative RT-PCR (qRT-PCR), these authors analyzed the expression profile of several maize *mTERF* genes under light and dark, and with salt and phytohormone treatments. Interestingly, the transcript levels of the maize *mTERF12* gene, the ortholog of Arabidopsis *mTERF6*, as well as those of maize *mTERF13* and *mTERF28,* changed after exposure to NaCl, AlCl_3_, or ABA compared to those of the untreated plants. This scenario supports the proposed function of *mTERF* genes in response to salinity [59].

#### 2.1.2. PPRs and Tolerance to Salt Stress

Like mTERFs, the PPR family of proteins participates in plant OGE, as PPRs regulate the processing of chloroplast and mitochondria RNAs, including splicing, stability, editing, and even translation [60]. As for mTERFs, the plant PPR family has also expanded, but to a higher level. Indeed, PPR is one of the largest families in land plants [61]. Accordingly, lycophyte Selaginella, rice, and Arabidopsis genomes, respectively, harbor more than 800, 650, and 450 *PPR* genes [62]. In contrast, metazoan genomes typically encode fewer than 10 PPR proteins [60]. PPRs are grouped into two subfamilies, namely P and PLS, according to the pattern of tandem repeats of a degenerate 35-amino acid repeat [63]. The PPR proteins of the P subfamily contain the canonical P motif, whereas those of the PLS subfamily contain, apart from the P motif, two variants that derive from the P motif: the large (L) and short (S) motifs [63]. The vast majority of PPR proteins are predicted to be targeted to mitochondria or chloroplasts. They can specifically bind RNA or DNA, and control different biological processes, such as embryogenesis, chloroplast, and seed development, as well as plastid retrograde signaling [47].

Despite the many PPR identified proteins in higher plants, very few have been reported to be involved in stress response: GENOMES UNCOUPLED 1 (GUN1) [64], PPR40 [45,46], ABA OVERLY-SENSITIVE 5 (ABO5) [65], LOVASTATIN INSENSITIVE 1 (LOI1) [66], PENTATRICOPEPTIDE REPEAT PROTEIN FOR GERMINATION ON NaCl (PGN) [47], SLOW GROWTH 1 (SLG1) [49], ABA HYPERSENSITIVE GERMINATION 11 (AHG11) [48], SLOW GROWTH 2 (SLO2) [50] and PPR96 [53] in Arabidopsis, and WHITE STRIPE LEAF (WSL) in rice [52]. All these PPR proteins are located in mitochondria, except for GUN1 and WSL, which are targeted to chloroplasts. Among these proteins, a role in salinity tolerance has been described for Arabidopsis PPR40, PGN, SLO2, PPR96, AHG11, and SLG1, and for rice WSL. Mitochondrial mutants *ppr40*, *pgn*, *ahg11, slg1*, and *slo2* and chloroplast mutant *wsl* display a similar response to ABA, salt, and osmotic stresses. They are all hypersensitive to these adverse environmental conditions during germination and early seedling growth. Most of these mutants exhibit changes in the endogenous levels of ROS, probably due to a perturbed mitochondrial function, which is the consequence of defects in the regulation of different mitochondrial RNA editing events in some cases.

The first PPR protein reported to be involved in salt tolerance was Arabidopsis PPR40. As mentioned above, the *ppr40-1* mutant is hypersensitive to ABA, salinity, and osmotic stress, accumulates high ROS levels, and shows an altered expression of stress-responsive genes and semidwarf growth (Table 1). No defects have been found in mitochondria RNA editing in the *ppr40-1* mutant [45]. A second mutant allele, *ppr40-2*, displays less severe alterations of developmental and stress responses, which suggests that is a weak loss-of-function allele of the *PPR40* gene [45]. The overexpression of the *PPR40* gene improves salt tolerance, likely because of the mitochondrial electron transport’s enhanced stability and reduced oxidative damage when faced with this adverse condition [46].

The Arabidopsis PGN protein is also targeted to mitochondria, and similarly to *ppr40* mutant seedlings, *pgn* mutants exhibit high ROS levels in response to salinity, as well as the down-regulation of different stress responsive genes (Table 1) [47]. Loss of PGN function results in altered responses to biotic and abiotic stresses. Thus, *pgn* mutants display susceptibility to necrotrophic fungal pathogens, as well as enhanced sensitivity to salinity, ABA, and glucose. These results suggest a role for PGN in the mitochondria homeostasis of ROS in response to biotic or abiotic stresses, including salinity [47].

The mutant *ahg11* was identified in a genetic screening, conducted to isolate Arabidopsis mutants with an altered response to ABA. To avoid the isolation of mutants affected in ABA synthesis or catabolism, EMS mutagenesis was performed in an *aba2-1* genetic background, which displayed low ABA levels. Mutants *ahg11* showed enhanced sensitivity to ABA, NaCl, and mannitol during seed germination, but not later during development, which indicates that *AHG11* functions in the stress response in both seeds and young seedlings [48]. RNA editing of the mitochondrial *nad4* transcript is lacking in mutant *ahg11*, which results in an amino acid change in the NAD4 protein of complex I (Table 1). Murayama et al. [48] suggest that this editing defect might presumably affect the activity of complex I and lead to a redox imbalance in mutant *ahg11* by causing altered responses to stress.

Like *ahg11*, the Arabidopsis *slg1* mutant shows similar ABA, salt, and osmotic stress phenotypes during germination and in early growth stages (Table 1). Unlike the AHG11 protein, SLG1 is also involved in stress tolerance after germination [49]. In this way, *slg1* adult plants are more tolerant to drought stress than the wild type, likely due to a more rapid stomata response in the mutant, which is consistent with greater sensitivity to ABA, as this hormone plays a key role in the response to drought stress by controlling stomata closure. Similarly to *ahg11*, the *slg1* mutant also shows defects in mitochondrial RNA editing, specifically in the *nad3* transcript, by bringing about a change from serine to proline in the NAD3 protein that strongly affects the structure of the protein, and concomitantly, the activity of complex I (Table 1) [49].

The *slo2* mutants are defective in an RNA editing factor that plays a key role in Arabidopsis growth through energy metabolism regulation [51]. Loss of *SLO2* function causes several RNA editing defects, and affects different mitochondrial transcripts and results in amino acid changes in four proteins belonging to complex I of the electron transport chain (Table 1). Interestingly, the levels of complexes I, III, and IV are substantially lower in *slo2* mutants. These results led Zhu et al. [51] to propose that RNA editing defects result in the dysfunction of mitochondrial electron transfer chain complexes, and contribute to the *slo2* mutant phenotype. In a later work, Zhu et al. [50] investigated the involvement of SLO2 in tolerance to stress. They found that *slo2* mutants display hypersensitivity to ABA, salt, and osmotic stress, and insensitivity to ethylene during germination and in early seedling stages (Table 1). On the contrary, adult *slo2* plants proved more drought- and salinity-tolerant. The *slo2* mutants also accumulate high H_2_O_2_ levels and are more susceptible to infection by the pathogenic fungus *Botrytis cinerea* (Table 1). Taken together, these results indicate that SLO2 is required for proper sensitivity to ABA, ethylene, biotic, and abiotic stress [50].

Recently, Liu and colleagues [53] characterized the Arabidopsis *PPR96* gene encoding a mitochondria-located protein, which is involved in the response to salt, ABA, and oxidative stress. The *ppr96* knockout mutants are more tolerant than the wild type to NaCl, ABA, and the oxidative stress caused by hydrogen peroxide (Table 1). *PPR96* expression was up-regulated in response to salt and oxidative stress treatments, according to a microarray in silico analysis, and was experimentally confirmed by qRT-PCR [53]. Nevertheless, Liu et al. [53] did not test whether the mutant displayed defects in the editing of mitochondrial transcripts.

Altogether, the phenotypic and molecular characterizations of mutants *ahg11, slg1*, and *slo2* strongly support a connection between OGE regulation in mitochondria at the level of RNA editing and plant tolerance to ABA and abiotic stresses, such as salinity.

A function performed by a chloroplast PPR protein in the abiotic stress response has been reported in rice [52]. The *wsl* mutant displays enhanced sensitivity to NaCl and ABA during germination, and accumulates higher ROS levels than the wild type (Table 1). Interestingly, plastid rRNAs and proteins accumulate in *wsl* plants at lower levels than in the wild type. This is likely the result of the defective plastid translation caused by the inefficient splicing of the chloroplast *rpl2* gene, which encodes the ribosomal protein RPL2 of this organelle [52]. Unlike the mitochondrial cases discussed above, Tan et al. [52] were unable to detect defects in chloroplast RNA editing in the *wsl* mutant, which indicates a role for this PPR protein in RNA regulation at the splicing rather than the editing level.

Very recently, the importance of PPR proteins in abiotic stresses tolerance has been further supported by a genome-wide transcriptomic analysis of the PPR family in poplar, which identified 154 *PtrPPR* genes induced by biotic and abiotic treatments, including salinity [67].

#### 2.1.3. Roles of Plant Organellar DEAD-Box RHs in Salinity Response

Another group of proteins involved in the control of OGE, which are hence experimentally related to the stress responses mediated by chloroplasts and plant mitochondria, is that of DEAD-box RNA helicases (DEAD-box RHs), the largest known RNA helicase subfamily. The helicase core of DEAD-box RHs is composed of the N and C terminal domains, which are structurally similar to recombination protein RecA, in which nine conserved motifs can be identified. Motif II of the N-terminal domain contains the amino acid sequence Asp-Glu-Ala-Asp (D-E-A-D), which gave a name to the subfamily. Variations in motif II determine three related RNA helicase subgroups, namely DEAD, DEAH, and Ski2, whose members are referred to as DExD/H-box proteins [68,69].

DEAD-box RHs are present in some viruses, many prokaryotes, and all eukaryotes, and they usually possess ATP-dependent RNA helicase activity, which allows them the localized unwinding of RNAs, and hence, to participate in different RNA metabolism processes, such as ribosome biogenesis, translation initiation, RNA splicing, turnover, and decay [70]. DEAD-box RHs are also involved in OGE in both chloroplasts and mitochondria. In fact, one of the first RH family members to be identified was Mss116, which is required for mitochondrial splicing in yeast [71].

Like mTERFs and PPRs, this family also extends in plants. Accordingly, Arabidopsis and rice show a similar number of presumed DEAD-box RHs, 58 and 60 [24], respectively, while *Drosophila melanogaster* and *Caenorhabditis elegans* show nearly half these numbers with 30 and 34 [72], respectively. By an in silico analysis, Nawaz and Kang [24] found that the numbers of chloroplastic DEAD-box RHs ranged from 7 to 12, and the mitochondrial ones from 4 to 7, in four different plant species.

Nowadays, growing evidence reveals the implication of DEAD-box RHs in plant responses to different kinds of stresses [24]. Regarding chloroplasts and mitochondria, it is known that some nuclear genes that encode organellar-localized DEAD-box RHs are induced by different stresses, including salinity. *HVD1* (*Hordeum vulgare* DEAD box protein1) was the first salt-responsive DEAD-box RH gene to be described encoding a chloroplast protein [73]. The expression of the *HVD1* gene is highly induced under salt and cold stresses, with subsequent recovery after exposure to salinity stress. In a genome-wide in silico study performed later, Umate et al. [74] found that several Arabidopsis DEAD-box RHs located in chloroplasts or mitochondria regulate transcription in response to various abiotic stresses (reviewed in [24]). In addition, the overexpression of some organellar-localized nuclear-encoded DEAD-box RHs has been described to confer resistance to salinity. Accordingly, the overexpression of OsSUV3 (*Oryza sativa* SUPPRESSOR OF VAR3), a mitochondrially-localized DExH/D-box-related DNA/RNA helicase, confers salt tolerance in rice by maintaining photosynthesis and antioxidant machinery [69]. Under salt stress, the levels of the plant hormones GA_3_ (gibberellic acid 3), zeatin, and IAA (indole-3-acetic acid) increase in the SUV3 overexpressing lines, which, in turn, may induce the expression of genes to help cope with stress [75]. Recently, the chloroplast-targeted DEAD-box RH BrRH22 of *Brassica rapa* has been described to be induced by several abiotic stresses, including salinity and ABA treatment. Likewise, the chloroplast-localized DEAD-box RH OsRH58 of rice is up-regulated by salinity, dehydration, and heat, but not ABA, unlike BrRH22 [76]. Transgenic Arabidopsis OsRH58-expressing plants show increased growth and yield seeds under no stress conditions. The heterologous expression of BrRH22 or OsRH58 in Arabidopsis improves germination and plant growth under salinity conditions. Both proteins may possess RNA chaperone activity, which may, in turn, influence plastid translation under stress conditions, as the Arabidopsis transgenic plants expressing BrRH22 or OsRH58 show larger amounts of some chloroplast genome-encoded proteins when treated with either NaCl or mannitol [76,77].

To our knowledge, Arabidopsis *AtRH3* is the only gene to encode an organellar-localized DEAD-box RH, whose involvement in the response to salinity has been reported by the isolation and characterization of loss-of-function mutant alleles. The impaired function of chloroplast targeted AtRH3 causes a range of effects, from embryonic lethality to delayed growth, as well as reduced greening of vegetative tissues, depending on the mutant allele’s strength [54]. Lee and colleagues [55] showed that young viable *atrh3-4* mutant seedlings were impaired in chloroplast biogenesis as these are smaller, and include fewer and smaller chlororibosomes than the wild type. The defective chloroplast development noted in *atrh3-4* seedlings gives rise to ABA-deficient and salt- and cold-sensitive phenotypes (Table 1) [55,56]. The function of AtRH3 is needed for the splicing of most intron-containing chloroplast genes [54,56] and the spliced/unspliced transcript ratio is lower in *atrh3* mutants (Table 1) [56]. Stress conditions do not affect this RNA processing defect, except for genes *ndhA* and *ndhB*, as the spliced/unspliced transcript ratio in *atrh3* mutants is significantly lower, especially under salt or cold stress, but not after dehydration stress [56]. The correct splicing of chloroplast transcripts is probably mediated by the RNA chaperone activity of AtRH3 [56]. More recently, loss of function mutations of *AtRH50*, another Arabidopsis gene that codes for a chloroplast-localized DEAD-box RH, has been shown to increase cold sensitivity as a result of the defective processing of chloroplastic rRNA, and hence inefficient plastid translation [78]. The response of *ath50* mutants to other abiotic stress, such as salinity, remains to be tested.

#### 2.1.4. Mutations in Plastid Ribosomal Protein PSRP2 Alter Salinity Tolerance

The previous sections noticeably illustrate the relationship between changes in the activity of nuclear genes involved in the post-transcriptional regulation of OGE at the RNA level and salt stress phenotypes. The involvement of plastidial translational machinery in plant salt responses has also been investigated. Accordingly, salt stress causes a transient suppression of the de novo synthesis of proteins in Arabidopsis suspension cell cultures [79]. Furthermore, Omidbakhshfard and colleagues [80] tested the expression by qRT-PCR of 170 genes related to protein synthesis in Arabidopsis leaves after NaCl exposure at different time points. Some genes showed up-regulation under salinity stress, and coded for chloroplast-located translation-related proteins, such as ribosomal proteins L11 (PRPL11) and L9-1 (also known as PIGGYBACK 2), ATAB2, an A/U-rich RNA-binding protein, which likely functions as an activator of translation, and PDF1B, a peptide deformylase required to remove the N-formyl group from nascent peptides. Interestingly, these genes have been previously described as being important for chloroplast development, and have been proposed to represent potential biotechnological targets for plant salt tolerance optimization [80].

As far as we know, only a salt-stress related phenotype has been described for a mutant affected in a plastid translational protein. Accordingly, the Arabidopsis *psrp2* mutant defective in PLASTID-SPECIFIC RIBOSOMAL PROTEIN 2 (PSRP2) of the 30S ribosomal subunit, one of the six Arabidopsis PSRP proteins, shows enhanced seedling growth under salinity stress (Table 1) [57]. On the contrary, the transgenic plants overexpressing PSRP2 display delayed germination and reduced seedling growth in response to salinity compared to the wild type (Table 1). These results suggest that PSRP2 functions as a negative regulator of germination and seedling growth under salinity conditions. Interestingly, PSRP2 contains two RNA recognition motifs (RRM), it can bind RNA and ssDNA, and it possesses RNA chaperone activity. All this suggests a connection between plastid translation and regulation of RNA metabolism [57].

### 2.2. Transcriptional Regulation of OGE and Plant Salt Tolerance

#### 2.2.1. Arabidopsis SIG5 Protects Chloroplasts from Abiotic Stress Damage 

Transcriptional regulation is also fundamental for OGE in plants, and mutations in the genes involved in this process can lead to a salt stress phenotype. Along these lines, a small family of nuclear genes, called *RpoT* (*RNA polymerase T7 phage-type*), encoding monomeric RNA polymerases, is responsible for the transcription of chloroplast or mitochondria genes [81]. Furthermore, the transcription of plastid genes requires a second type of DNA-dependent RNA polymerase, similar to those of prokaryotes, which is encoded by the organellar genome and dubbed PEP (plastid-encoded RNA polymerase). PEP comprises four catalytic core subunits (α, β, β’, and β’’, respectively, encoded by plastid genes *rpoA, rpoB, rpoC1*, and *rpoC2*) and requires nucleus-encoded sigma factors (SIG) to recognize specific promoter sequences to initiate plastid gene transcription [82].

One of the six *SIG* Arabidopsis genes, *SIG5*, is up-regulated in response to different stress conditions, including salinity [58]. SIG5 is required for the transcriptional activation of the blue light-responsive promoter of the *psbD* gene that encodes photosystem II (PSII) reaction center protein D2, which binds the essential redox co-factors needed for photosynthetic electron transfer [58]. Besides, seed germination and recovery from damage of PSII after salt stress are delayed in loss-of-function mutant *sig5-2* compared to the wild type (Table 1). This led Nagashima et al. [58] to propose that SIG5 enhances the repair of the PSII reaction center under stress by protecting chloroplasts, and hence, the plants exposed to adverse environmental stress conditions. Interestingly, the expression of the orthologous gene from the liverwort *Marchantia polymorpha* (*MpSIG5*) is induced by blue-light irradiation under several stress conditions, which suggests the conservation of the responsible mechanism [83]. Nevertheless, the expression of the *psbD* gene of Marchantia did not occur in conjunction with *MpSIG5* induction, which indicates that SIG5 might play a divergent physiological role in different plant phyla [83].

Recently, Zhao et al. [84] obtained Arabidopsis *SIG5* overexpression lines, which are more tolerant to salt stress than the wild type. These results are in agreement with those from Nagashima et al. [58] mentioned above. Furthermore, Zhao and colleagues [84] also identified an upstream regulator of *SIG5*, ATHB17 (ARABIDOPSIS THALIANA HOMEOBOX 17), an Arabidopsis HD-Zip transcription factor that binds to the *cis*-elements present in the *SIG5* promoter. Similarly to *SIG5, ATHB17* loss-of-function reduced plant salt tolerance, whereas ATHB17 overexpression enhanced it. Interestingly, these phenotypes were at least partially dependent on SIG5. Accordingly, *ATHB17* overexpression in a *sig5-1* mutant background showed tolerance to a salinity intermediate between that of the *ATHB17* overexpression lines and the *sig5-1* mutant [84]. Under salt stress conditions, various plastid genes regulated by SIG5 were down-regulated in the *ATHB17* overexpression lines, but were up-regulated in the *ATHB17* knockout lines, which suggests the positive regulation of these genes through SIG5 [84].

## 3. Conclusions and Future Perspectives

**Figure 1:**
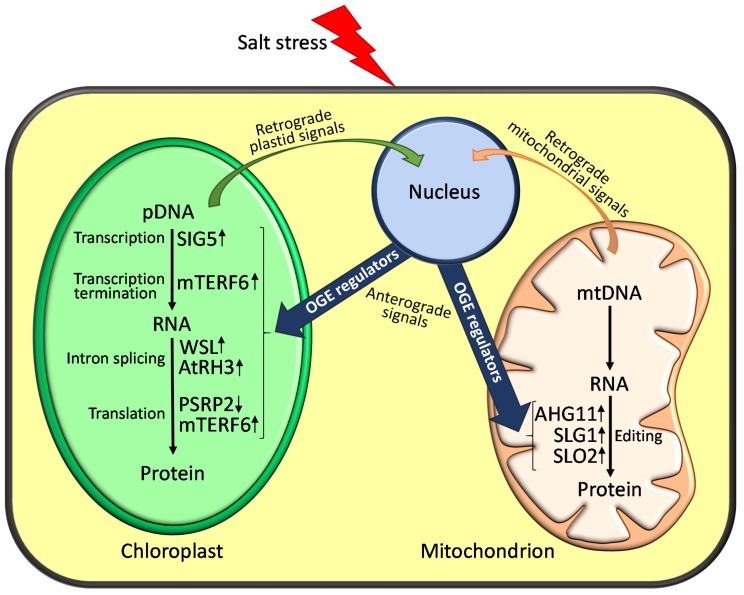
Schematic representation of the cellular functions of OGE regulators in plant response to salinity. Salt stress can perturb chloroplast or mitochondrion homeostasis and this would be communicated to the nucleus through retrograde signals, leading to changes in nuclear gene expression. In turn, this would activate the expression of OGE regulators (e.g., mTERFs, PPRs or DEAD-box RHs proteins), which would result in anterograde signalling responses to adjust organellar function to salinity. In the figure, inside a mitochondrion (purple) and chloroplast (green), only those molecularly-characterized OGE regulators whose mutations lead to altered responses to salinity are depicted. The OGE processes affected in these mutants, as well as their enhanced (↑) or reduced (↓) sensitivity to salt stress, are shown (see also Table 1 for further information).

The results compiled in this review reveal that accurate OGE regulation in chloroplasts and mitochondria is fundamental for plants to tolerate and adapt to adverse environmental conditions, such as salt stress (Figure 1). Consequently, perturbed OGE homeostasis affects plant responses to salinity, which can be detrimental for their survival. However, we are still far from fully understanding the role that OGE regulation plays in adapting plants to salinity. To make progress, it is necessary to know more details of the molecular functions of several genes involved in the control of OGE, whose disturbance results in stress phenotypes. This is especially relevant for the *mTERF* genes related to stress tolerance, because the mechanistic insight for most of them is lacking. Furthermore, unraveling why particular stressors specifically affect the expression of some OGE-related genes would shed light on their functional roles in plant tolerance to abiotic stresses. If we consider that a stress mutant phenotype has been reported for only a few *PPR*, *mTERF*, or *DEAD-box RH* genes, it is fundamental to identify and characterize mutants in plant model systems and crops affected in novel genes that transcriptionally or post-transcriptionally regulate OGE in plastids and mitochondria. This information is expected to contribute to the comprehensive understanding of the mechanisms regulating OGE in plants, and to provide insights to improve stress tolerance by identifying potential biotechnological targets for better plant growth and crop yields under salinity conditions.

## Figures and Tables

**Table 1:** Mutants affected in OGE displaying a salt stress phenotype.

Mutant	Species	Organelle	Affected Gene (AGI code)	Mutant Stress Phenotype	Molecular Function	Reference
*mda1*	*Arabidopsis thaliana*	Chloroplast	*mTERF5* (AT4G14605)	Reduced sensitivity to ABA, salt, and osmotic stress; altered sugar responses.		[40]
*mterf6*	*Arabidopsis thaliana*	Chloroplast	*mTERF6* (AT4G38160)	Salt- and ABA-hypersensitive.	Maturation of the chloroplast isoleucine tRNA (*trnI.2*) gene; transcription termination of the plastid *rpoA* polycistron	[35,36,42]
*mterf9*	*Arabidopsis thaliana*	Chloroplast	*mTERF9* (AT5G55580)	Reduced sensitivity to ABA, salt, and osmotic stress; altered sugar responses.		[41]
*mterf10*	*Arabidopsis thaliana*	Chloroplast	*mTERF10* (AT2G34620)	Hypersensitive to salt stress; *mTERF10* overexpression leads to enhanced salt and ABA tolerance.		[43]
*mterf11*	*Arabidopsis thaliana*	Chloroplast	*mTERF11* (AT3G18870)	Hypersensitive to ABA and salt stress. *mTERF11* overexpression leads to enhanced salt tolerance and increased ABA sensitivity.		[43]
*ppr40*	*Arabidopsis thaliana*	Mitochondria	*PPR40* (AT3G16890)	Enhanced sensitivity to salt, ABA, and oxidative stress. *PPR40* overexpression improves salt tolerance and reduces oxidative stress.		[45,46]
*pgn*	*Arabidopsis thaliana*	Mitochondria	*PGN* (AT1G56570)	Hypersensitive to salt, ABA, and glucose. Enhanced ROS levels under salt stress.		[47]
*ahg11*	*Arabidopsis thaliana*	Mitochondria	*AHG11* (AT2G44880)	Hypersensitive to ABA, salt, and osmotic stress. Enhanced ROS levels.	*nad4* mitochondrial RNA editing	[48]
*slg1*	*Arabidopsis thaliana*	Mitochondria	*SLG1* (AT5G08490)	Hypersensitive to ABA, salt, and osmotic stress. Enhanced tolerance to drought in adult plants.	*nad3* mitochondrial RNA editing	[49]
*slo2*	*Arabidopsis thaliana*	Mitochondria	*SLO2* (AT2G13600)	Hypersensitive to ABA, salt, and osmotic stress. Insensitive to ethylene. Enhanced tolerance to drought and salt in adult plants. Enhanced ROS levels.	*nad4L*, *nad7*, *mttB*, and *nad1* mitochondrial RNA editing	[50,51]
*wsl*	*Oryza sativa*	Chloroplast	*WSL* (LOC_Os01g37870)	Hypersensitive to ABA, salinity, and sugar. Enhanced H_2_O_2_ levels.	Splicing of chloroplast transcript *rpl2*	[52]
*ppr96*	*Arabidopsis thaliana*	Mitochondria	*PPR96* (AT2G03380)	Reduced sensitivity to ABA, salt, and oxidative stress.		[53]
*atrh3*	*Arabidopsis thaliana*	Chloroplast	*AtRH3* (AT5G26742)	ABA-deficient and salt-sensitive.	Splicing of most intron-containing chloroplast genes	[54,55,56]
*psrp2*	*Arabidopsis thaliana*	Chloroplast	*PSRP2* (AT3G52150)	Enhanced tolerance to salt, osmotic, and cold stress. *PSRP2* overexpression leads to reduced tolerance to salt, dehydration, and cold stress.		[57]
*sig5*	*Arabidopsis thaliana*	Chloroplast	*SIG5* (AT5G24120)	Hypersensitive to salt stress.	Repair of stress-damaged PSII through the transcriptional activation of the *psbD* blue light receptor	[58]

## Abbreviations

ABA	abscisic acid
DEAD-box RHs	DEAD-box RNA helicases
mTERF	mitochondrial transcription termination factor
OGE	organellar gene expression
PPR	pentatricopeptide repeat proteins
PSRP2	PLASTID-SPECIFIC RIBOSOMAL PROTEIN 2
RRM	RNA-recognition motifs (RRMs)
SIG5	SIGMA FACTOR 5
